# WTD Attenuating Rheumatoid Arthritis *via* Suppressing Angiogenesis and Modulating the PI3K/AKT/mTOR/HIF-1α Pathway

**DOI:** 10.3389/fphar.2021.696802

**Published:** 2021-09-27

**Authors:** Xin Ba, Ying Huang, Pan Shen, Yao Huang, Hui Wang, Liang Han, Wei Ji Lin, Hui Jia Yan, Li Jun Xu, Kai Qin, Zhe Chen, Sheng Hao Tu

**Affiliations:** ^1^ Institute of Integrated Traditional Chinese and Western Medicine, Tongji Hospital, Tongji Medical College, Huazhong University of Science and Technology, Wuhan, China; ^2^ Department of Integrated Traditional Chinese and Western Medicine, Tongji Hospital, Tongji Medical College, Huazhong University of Science and Technology, Wuhan, China; ^3^ Rehabilitation and Sports Medicine Research Institute of Zhejiang Province, Zhejiang Provincial People’s Hospital, People’s Hospital of Hangzhou Medical College, Hangzhou, China

**Keywords:** rheumatoid arthritis, fibroblast-like synoviocytes, wutou decoction, angiogenesis, collagen-induced arthritis, network pharmacology

## Abstract

**Background:** Wutou Decoction (WTD), as a classic prescription, has been generally used to treat rheumatoid arthritis (RA) for two thousand years in China. However, the potential protective effects of WTD on rheumatoid arthritis and its possible mechanism have rarely been reported.

**Purpose:** The aim of this study was to explore the possible mechanism of WTD against RA and a promising alternative candidate for RA therapy.

**Methods:** A model of collagen-induced arthritis (CIA) was constructed in rats to assess the therapeutic effects of WTD. Histopathological staining, immunofluorescence, and western blotting of synovial sections were conducted to detect the antiangiogenic effects of WTD. Then, cell viability assays, flow cytometry, scratch healing assays, and invasion assays were conducted to explore the effects of WTD on MH7A human fibroblast-like synoviocyte (FLS) cell proliferation, apoptosis, migration, and invasion *in vitro*. The ability of WTD to induce blood vessel formation after MH7A cell and human umbilical vein endothelial cell line (HUVEC) coculture with WTD intervention was detected by a tube formation assay. The mechanisms of WTD were screened by network pharmacology and confirmed by *in vivo* and *in vitro* experiments.

**Results:** WTD ameliorated the symptoms and synovial pannus hyperplasia of CIA rats. Treatment with WTD inhibited MH7A cell proliferation, migration, and invasion and promoted MH7A apoptosis. WTD could inhibit MH7A cell expression of proangiogenic factors, including VEGF and ANGI, to induce HUVEC tube formation. Furthermore, the PI3K-AKT-mTOR-HIF-1α pathway was enriched as a potential target of WTD for the treatment of RA through network pharmacology enrichment analysis. Finally, it was confirmed *in vitro* and *in vivo* that WTD inhibits angiogenesis in RA by interrupting the PI3K-AKT-mTOR-HIF-1α pathway.

**Conclusion:** WTD can inhibit synovial hyperplasia and angiogenesis, presumably by inhibiting the migration and invasion of MH7A cells and blocking the production of proangiogenic effectors in MH7A cells. The possible underlying mechanism by which WTD ameliorates angiogenesis in RA is the PI3K-AKT-mTOR-HIF-1α pathway.

## Introduction

Rheumatoid arthritis (RA) is a chronic autoimmune disease with the characteristic pathological changes of persistent synovitis, hyperplastic synovial pannus tissue formation, the destruction of cartilage and bone, and the presence of autoantibodies (especially rheumatoid factor and citrullinated peptide) (Mcinnes and Schett, 2011). Multiple genetic factors (e.g., HLA-DRB1 and PTPN22) and environmental risks (e.g., smoking) are closely related to the development of RA (Smolen et al., 2016). RA affects approximately 1% of the global population, especially elderly individuals and women, with 5–50 per 100,000 individuals newly diagnosed with RA annually (Myasoedova et al., 2010; Tobon et al., 2010). In recent decades, disease-modifying antirheumatic drugs (DMARDs) have been the predominant therapeutic agents for RA. The leading DMARD is methotrexate, which is widely accepted as a first-line regimen due to its superior efficacy and economic advantages. Biological agents and small molecule targeted drugs are used when RA cannot be controlled or toxic side effects arise with DMARD treatment (Smolen et al., 2017; Sparks et al., 2021). However, biological targeted DMARDs are expensive and may increase the risk of infection (Burmester and Pope, 2017). More than 30% of patients still cannot satisfactorily control this disease after using the drugs mentioned above (Singh et al., 2015; Favalli et al., 2017; Aletaha and Smolen, 2018). Therefore, it is necessary to find new drugs or therapeutic strategies for RA patients.

Angiogenesis in synovial tissue is considered an important early event in the development of RA (Elshabrawy et al., 2015). Angiogenesis is mediated by proangiogenic factors and the corresponding receptors on the surface of endothelial cells, including growth factors, proinflammatory cytokines, chemokines, and others (Latacz et al., 2020). In particular, hypoxia-inducible factor-1 (HIF-1) and vascular endothelial growth factor (VEGF) are considered major mediators of angiogenesis. In an inflammatory or hypoxic environment, the expression of HIF-1 increases to mediate angiogenesis. HIF-1 is a transcription factor composed of constitutive expression of the β subunit and oxygen-regulated α subunit, which mainly determines HIF-1 activation. HIF-1α is quickly degraded under normal oxygen conditions but stabilized under hypoxic conditions when it quickly translocates to the nucleus, where it induces the expression of VEGF. VEGF then promotes the activation of endothelial cells to induce inflammation and thereby establish crosstalk between vascular production and articular inflammation in RA (Ferrara, 2004). An increase in the number of blood vessels can provide more oxygen and nutrients to synovial tissue, but abnormal vessels facilitate inflammatory cell migration and pannus formation, thus destroying neighboring cartilage and bones, as observed in RA (Paleolog and Miotla, 1998). Fibroblast-like synoviocytes (FLSs) are a key component of the proliferative synovial membrane and play a crucial role in the pathogenesis of RA (Nygaard and Firestein, 2020). Notably, proangiogenic factors are mainly produced by RA synovial tissue macrophages and fibroblasts. The RA synovial tissue lining layer plays an indispensable role in maintaining synovitis through neovascularization. A recent study indicated that RA-FLSs are closely involved in synovial angiogenesis (Wei et al., 2020). Therefore, modulation of the angiogenesis induced by FLSs is a promising strategy for the treatment of RA (Bottini and Firestein, 2013).

Wutou decoction (WTD), a traditional Chinese medicine (TCM), has been used to treat RA for two thousand years in China, and it has been gradually accepted by clinicians due to its excellent efficacy and few side effects (Moudgil and Berman, 2014). WTD contains five traditional drugs, including *Glycyrrhizae Radix Preparata*, *Ephedrae Herba*, *Paeoniae Radix Alba*, *Aconiti Radix Cocta*, and *Astragali Radix*. Modern pharmacological studies have shown that WTD has anti-inflammatory effects, regulates immunity, relieves pain, and has antiangiogenic effects (Lu et al., 2015; Guo et al., 2020). However, the underlying mechanism by which WTD alleviates RA is still unknown, which impedes its further clinical application.

In the past, due to the multiple components, targets, and channels that are characteristic of TCM compounds, their mechanisms of action have been difficult to clarify (Yuan et al., 2020). Network pharmacology is considered a promising method for complicated mechanistic studies and new drug discovery from TCMs (Tao et al., 2013). TCM network pharmacology methods establish a drug-gene-disease network in combination with existing clinical drug targets to predict the target spectrum and pharmacological effects of herbal compounds and to explain the combination rules and network regulation of herbal formulas (Guo et al., 2017). Therefore, we carried out this study based on network pharmacology to investigate the mechanism by which WTD ameliorates angiogenesis in RA.

## Materials and Methods

### Preparation of Wutou decoction

WTD contains five traditional drugs, including 6 g of *Aconiti Radix* (*Aconitum carmichaeli* Debeaux.), 9 g of *Ephedrae Herba* (*Ephedra sinica* Stapf), 9 g of *Paeoniae Radix Alba* (*Paeonia lactiflora* Pall.), 9 g of *Astragali Radix* (*Astragalus mongholicus* Bunge), and 9 g of *Glycyrrhiza Radix Preparata* (*Glycyrrhiza uralensis* Fisch. ex DC.). The composition of WTD is shown in Table 1. The herbs were purchased from Tongji Hospital Pharmacy Department (Wuhan, China). WTD was prepared as previously described (Zhang et al., 2015). All herbs were soaked in 2 L of pure water for 2 h in advance. First, *Aconiti Radix Cocta* was boiled for 0.5 h. Then, the remaining drugs were added and boiled for 0.5 h. The decoction was filtered through a strainer, and the filtrate was collected. The herbs were boiled for 0.5 h with 2 L of water, and the filtrates were extracted following the above method. The filtrates were combined and concentrated to a concentration of 0.75 g/ml. For *in vitro* experiments, the pH of the decoction was adjusted to neutral, the penetrating pressure of the decoction was adjusted to 310 mmol/L, and then the freeze-dried powder was condensed. Subsequently, the filtrate was filtered through 0.22 μm sterile filters, and this filtrate was collected and stored at 4°C for no more than 1 week. Finally, the decoction was diluted to the appropriate concentration with saline for the *in vivo* experiments and with PBS for the *in vitro* experiments before use.

**TABLE 1:** The composition of Wutou decoction (WTD)

Herbal medicine	Chinese name	Plant part	Occupied percent	Week usage (g)
Aconiti Radix	Chuanwu	Root	14.28%	24
Ephedra Herba	Mahuang	stems	21.43%	36
Paeoniae Radix Alba	Baishao	Root	21.43%	36
Hedysarum Multijugum Maxim	Huangqi	Root	21.43%	36
Glycyrrhiza Radix Preparata	Gancao	Root	21.43%	36

### High-Performance Liquid Chromatography (HPLC) Fingerprinting of Wutou decoction

HPLC fingerprint analysis was used to identify the main chemical components of the WTD extract. The extract was dissolved in water at a concentration of 1.92 g/ml (w/v) and further diluted to 0.96 g/ml (w/v) with methanol-water (50:50). Samples were passed through an Acclaim™ 120 C18 column (4.6 mm × 250 mm, 5 μm) at a flow rate of 1.0 ml/min with the mobile phases of methanol (A) and 0.1% phosphoric acid (B). HPLC signals were measured at a detection wavelength of 240 nm with gradient elution as detailed in Table 1. Ephedrine hydrochloride, pseudoephedrine hydrochloride, paeoniflorin, verbasil-7-O-glucoside, glycyrrhizin, benzoylaconitine, benzoylneoaconitine, benzoylhypoaconitine, and glycyrrhizinate were purchased from Chengdu MUST Biotechnology Co., Ltd. (Chengdu, China). The TCM compound solution was evenly mixed by vortexing, 200 µL of the solution was accurately measured, and 1 ml of methanol:water (8:2, V/V) was added, and the mixture was vortexed. After centrifugation for 10 min at 4°C and 20,000×g, the supernatant was filtered through a 0.22 µm filter membrane and the filtrate was collected for analysis.

### Construction of a Collagen-Induced Arthritis Rat Model and Wutou decoction Treatment

Fifty male Wistar rats (6 weeks of age, 160–200 g) were provided by the Hubei Provincial Centers for disease Control and Prevention (Animal Certificate of Conformity: SCXK (Hubei) 2015–0,018). Rats were kept under normal illumination conditions, and the room temperature was maintained at 22 ± 4°C. Bovine type II collagen (Chondrex Inc. Redmond, WA, United States of America) was mixed with complete Freund’s adjuvant (Sigma, United States of America) at a proportion of 1:1. Each rat was injected with 0.3 ml of the emulsion at the end of the tail and on the back, and a second injection was administered on the seventh day. The study program was approved by the Ethical Committee of Tongji Hospital, Tongji Medical College, Huazhong University of Science and Technology (TJ-A20170502).

The level of arthritis in the rats was graded with the following evaluation system, and the highest grade was 16 points. The arthritis severity was expressed with a visual semiquantitative score system for each paw from 0 to 4 according to the following criteria: 0, normal joints; 1, slight swelling or a digital red spot; 2, red skin and slight swelling in the ankles and feet; 3, moderate swelling and erythema; and 4, severe swelling and erythema involving the entire posterior claw or precipitation (Kollias et al., 2011). On day 14, successfully modelled rats with arthritis scores over 6 were randomly allocated to 4 groups: the Control, the CIA, WTD at low-dose (WTD-L), and WTD at high-dose (WTD-H) groups according to a random number table. Treatment was given daily for 28 days from day 14 to day 42. According to the human adult (body weight of 70 kg) dosage of 42 g/d (the conversion factor used for conversion of human to rat is 6), the dosage of WTD (3.75 and 7.5 g/kg/day) was selected for the rats. This low dosage of WTD is nearly equivalent to the daily RA patient dosage. The WTD groups were given intragastric with corresponding doses of WTD (3.75 and 7.5 g/kg/day). The control group and the model group were given intragastric saline. The animals were sacrificed with 2% pentobarbital on day 42. Blood, knee synovial tissues, and hind paw ankles were excised from the rat.

### Cell Viability Assay

The human FLS cell line MH7A (RIKEN Cell Bank, Japan) was incubated in sterile DMEM supplemented with 10% FBS, 100 U/mL penicillin, and 80 U/mL streptomycin. MH7A cells (1×10^4^ cells/mL) were seeded in 96-well plates and incubated in sterile DMEM with different concentrations of WTD (1 or 10 mg/ml) for 24, 36, or 48 h. Cell viability was determined by the 3-(4,5-dimethyl-2-thiazolyl)-2,5-diphenyl-2 H-tetrazolium bromide (MTT) method. The OD value was measured at a wavelength of 570 nm with a microplate reader (United States of America, Bio-Tek), and the inhibition rate was calculated. Three independent experiments were performed.

### Cell Apoptosis Analysis by Flow Cytometry

MH7A cells were seeded in 6-well plates and incubated with different concentrations of WTD (1 or 10 mg/ml) for 24 h. Adherent MH7A cells were digested with trypsin without EDTA. The cells were resuspended in 200 μL of 1× binding buffer in a centrifuge tube, and the concentration of cells was adjusted to 1×10^6^ cells/ml. Then, apoptosis was detected with an Annexin V-PE/7-AAD staining kit (BD Biosciences) and assayed by flow cytometry (BD FACS caliber; BD Biosciences). Data analysis was conducted using FlowJo software.

### Scratch Healing Assay

MH7A cells were seeded in a 12-well plate (5 × 10^5^ cells in 200 µL of DMEM per well). Wounds were made using a pipette tip at time 0 h. After washing with PBS, DMEM containing 5% FBS with or without TNFα (40 ng/ml) and different concentrations of WTD (1 or 10 mg/ml) were added to the wells. The cells were placed in a 37°C, 5% CO_2_ incubator for culture. Then, photographs of the wells were taken immediately at 0 h and at 24 and 48 h, and the distance and area of each scratch was measured. Three independent experiments were performed.

### Invasion Assay

The upper surfaces of Transwell inserts were prepared with Matrigel (1.25 mg/ml, 20 µL/well) for 45 min at 37°C. All groups except the blank group were treated with or without TNFα (40 ng/ml) and different concentrations of WTD (1 or 10 g/ml) for 24 h at 37°C and 5% CO2. After 24 h of incubation, the noninvasive cells on the upper membrane surface were removed by wiping with a cotton swab. The cells were stained with crystal violet staining solution and photographed under a phase-contrast microscope at 200 × magnification. Five random fields were imaged, and the experiment was repeated three times.

### Tube Formation Assay

To examine the inhibitory effects of WTD on MH7A-induced HUVEC tube formation, a tube formation assay was performed as described previously. Matrigel (10 mg/ml, 35 µL/well) was spread on the bottom of a 96-well plate and placed in a 37°C incubator for 45 min. After it solidification, Matrigel was added (10 mg/ml, 15 µL/well) to cover the bottom of the 96-well plate to ensure that the liquid was level, and the plate was placed in a 37°C incubator overnight. Human umbilical vein endothelial cells (HUVECs) were inoculated on the bottom of a 24-well plate, and 600 µL of DMEM containing 10% serum was added. After digestion of the MH7A cells with or without TNFα (40 ng/ml) and different concentrations of WTD (1 or 10 g/ml) for 24 h in advance, the HUVECs were seeded on the upper chamber surface of a 24-well Transwell chamber, and 200 µL of DMEM containing 10% serum was added. HUVECs and FLSs were cocultured for 24 h at 37°C with 5% CO_2_. Then, HUVECs were seeded on 96-well plates with Matrigel at a density of 2 × 10^4^ cells per well. After incubation for 6 h, the capillary tube formation of each well was imaged with a phase-contrast microscope. Quantitation of the antiangiogenic activity of WTD on tube formation was performed by counting the number of branch points. Three independent assays were performed.

### Immunohistochemical and Immunofluorescence Staining

Synovial tissue was fixed in 4% paraformaldehyde and embedded in paraffin. It was then heated in an oven at 60°C for 1 h, dewaxed in dimethyl benzene, polarized with descending concentrations of alcohol (100, 95, and 75%), and washed with water. Endogenous peroxidase activity was prevented by incubation at room temperature with a 3% H2O2 solution. The antigenic epitope was blocked by incubation with 20% normal goat serum for 1 h followed by incubation with primary antibodies at 4°C for 12 h. After washing with water three times, the slides were incubated with a HRP-conjugated secondary antibody for 1 h at room temperature. Then, the slides were visualized with DAB, and counterstaining was performed with hematoxylin. Images were taken with an Olympus fluorescence microscope system, with at least 5 random overlaps of each portion.

For immunofluorescence staining, preparation of the slides followed the same method as that for IHC. However, in contrast, an Alexa powder 488- or 594-conjugated secondary antibody (1:1,000) was used instead of the HRP-conjugated secondary antibody, and nuclei were dyed using a DAPI solution. There was no need for visualization with DAB, and counterstaining was performed with hematoxylin.

### Western Blot

MH7A cells were pretreated with WTD for 24 h and subsequently treated with or without TNFα (40 ng/ml) and different concentrations of WTD (1 or 10 g/ml). Total protein was extracted from the synovial tissue, and the protein concentration was quantified with a bicinchoninic acid protein assay kit. Each protein sample (30 μg/lane) was separated by 8–15% SDS-PAGE (80 V, for 1 h) and then transferred to a 0.22 μm nitrocellulose filter membrane (280 mA, 1 kD/min). The membrane was blocked with 5% skim milk for 1 h at room temperature and incubated overnight at 4°C with primary antibodies against β-actin, PI3K, AKT, p-AKT, mTOR, p-mTOR, VEGF, ANG1, VEGFR2, TEK (1:1,000, ABclonal, China), and HIF-1α (1:1,000, CST, United States of America). Subsequently, the membranes were incubated with secondary antibiotics at room temperature for 1 h. Finally, the membranes were visualized with an Odyssey Infrared Imaging system (LI-COR Biosciences, United States of America). The images was quantified using ImageJ software and standardized against β-actin. Three independent assays were performed.

### Network Pharmacology-Based Analysis of Wutou decoction

All chemical compounds from the five herbs in WTD were obtained from the TCMSP database (https://tcmspw.com/tcmsp.php) (Ru et al., 2014) and the Bioinformatics Analysis Tool for Molecular Mechanism of Traditional Chinese Medicine (BATMAN-TCM, http://bionet.ncpsb.org/batman-tcm/) (Liu et al., 2016). Before target prediction, the candidate components should meet the following parameters: oral bioavailability (OB) < 30% and drug-likeness (DL) < 0.18 to obtain compounds with higher oral absorption, utilization, and biological characteristics. Then, the compounds from multiple databases were combined, and duplicated items were removed. The target proteins were converted into genes *via* UniProt (http://www.UniProt.org/).

For disease target identification, RA-related target proteins were screened from the DrugBank (Wishart et al., 2018), OMIM (Amberger et al., 2015), pharmGK (Barbarino et al., 2018), Therapeutic Target database (Wang et al., 2020), DisGeNET (Pinero et al., 2017) and GeneCards (Stelzer et al., 2016) databases by using the keyword “RHEUMATOID ARTHRITIS”. The overlapping WTD targets and RA targets were considered WTD-regulated RA targets (Supplementary Table S1). The “compound-target” network was established with Cytoscape 3.8 software. The 348 overlapping target genes were considered potential targets of WTD for the treatment of RA and were used to construct a PPI network. Gene ontology (GO) and Kyoto Encyclopedia of Genes and Genomes (KEGG) pathway enrichment analysis of the WTD-regulated RA target network was conducted by the Metascape database (http://metascape.org/gp/index.html) (Zhou et al., 2019). Finally, the KEGG pathway map was drawn by using KEGG mapper (https://www.genome.jp/kegg/) (Kanehisa et al., 2017).

### Statistical Analysis

Statistical analyses were performed using GraphPad Prism 8.0. Data from multiple repeat experiments are presented as the means ± standard deviation (SD). Significant differences among the groups were evaluated by one-way analysis of variance (ANOVA) and Dunnett’s *t*-test, and *p* < 0.05 was considered statistically significant. The number of replicates and/or the total number of animals are shown in the figure legends or within the figures.

## Results

### High-Performance Liquid Chromatography Fingerprinting of Wutou decoction

**FIGURE 1:**
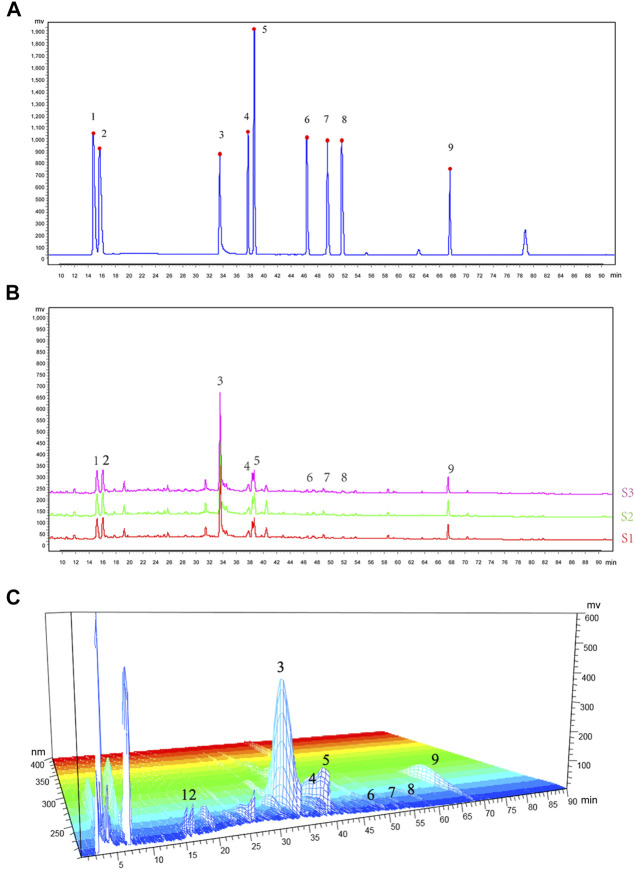
HPLC fingerprinting of WTD. **(A)** HPLC fingerprinting chromatograms of the reference standards. **(B)** HPLC fingerprinting chromatograms of the WTD extracts. In the chromatograms the peaks are labeled as follows: 1) ephedrine hydrochloride; 2) pseudoephedrine hydrochloride; 3) paeoniflorin; 4) verbasil-7-O-glucoside; 5) glycyrrhizin; 6) benzoylaconitine; 7) benzoylneoaconitine; 8) benzoylhypoaconitine; and 9) glycyrrhizinate. **(C)** HPLC 3D fingerprinting chromatograms of the WTD extracts.

A total of nine chemical components in WTD were determined, including benzoylaconitine (CW), benzoylneoaconitine (CW), benzoylhypoaconitine (CW), ephedrine hydrochloride (MH), pseudoephedrine hydrochloride (MH), paeoniflorin (BS), verbasil-7-O-glucoside (HQ), glycyrrhizinate (GC), and glycyrrhizin (GC). The HPLC fingerprint results of the standard are shown in Figure 1A and the sample is shown in Figure 1B. Three different batches of medicinal materials were repeated to confirm that these nine substances could be stably detected. The relative content of each substance in WTD was calculated and the detection of nine compounds in different wavelength ranges were better shown by 3D HPLC fingerprint (Figure 1C). The retention times and contents of the main chemical components of WTD are shown in Table 2.

**TABLE 2:** HPLC fingerprinting of WTD

Herb name	Main chemical composition	RT (min)	Sample RT (min)			Constent estimation (µg/ml)		
			S1	S2	S3	S1	S2	S3
Aconiti Radix Cocta	Benzoylaconitine	49.621	48.941	48.958	48.917	88.2295	95.72827	95.94493
	Benzoylneoaconitine	46.532	46.554	46.567	46.531	37.71674	41.45507	41.23415
	Benzoylhypoaconitine	51.761	51.845	51.864	51.817	46.46893	51.20894	51.51884
Ephedrae Herba	Ephedrine Hydrochloride	15.190	15.188	15.181	15.18	429.4898	467.1652	483.6357
	Pseudoephedrine hydrochloride	16.071	16.06	16.053	16.05	351.8483	394.7957	402.0504
Paeoniae Radix Alba	Paeoniflorin	33.535	33.557	33.558	33.531	3341.241	3688.472	3739.668
Astragali Radix	Verbasil-7-0-glucoside	37.697	37.798	37.823	37.785	226.2418	236.1833	237.9742
Glycyrrhiza Radix Preparata	Glycyrrhizinate	67.544	67.542	67.566	67.546	522.187	579.8064	587.9906
	Glycyrrhizin	38.623	38.631	38.652	38.616	92.3727	100.6486	101.4226

### Wutou decoction Treatment Ameliorates Arthritis in Collagen-Induced Arthritis Rats

**FIGURE 2:**
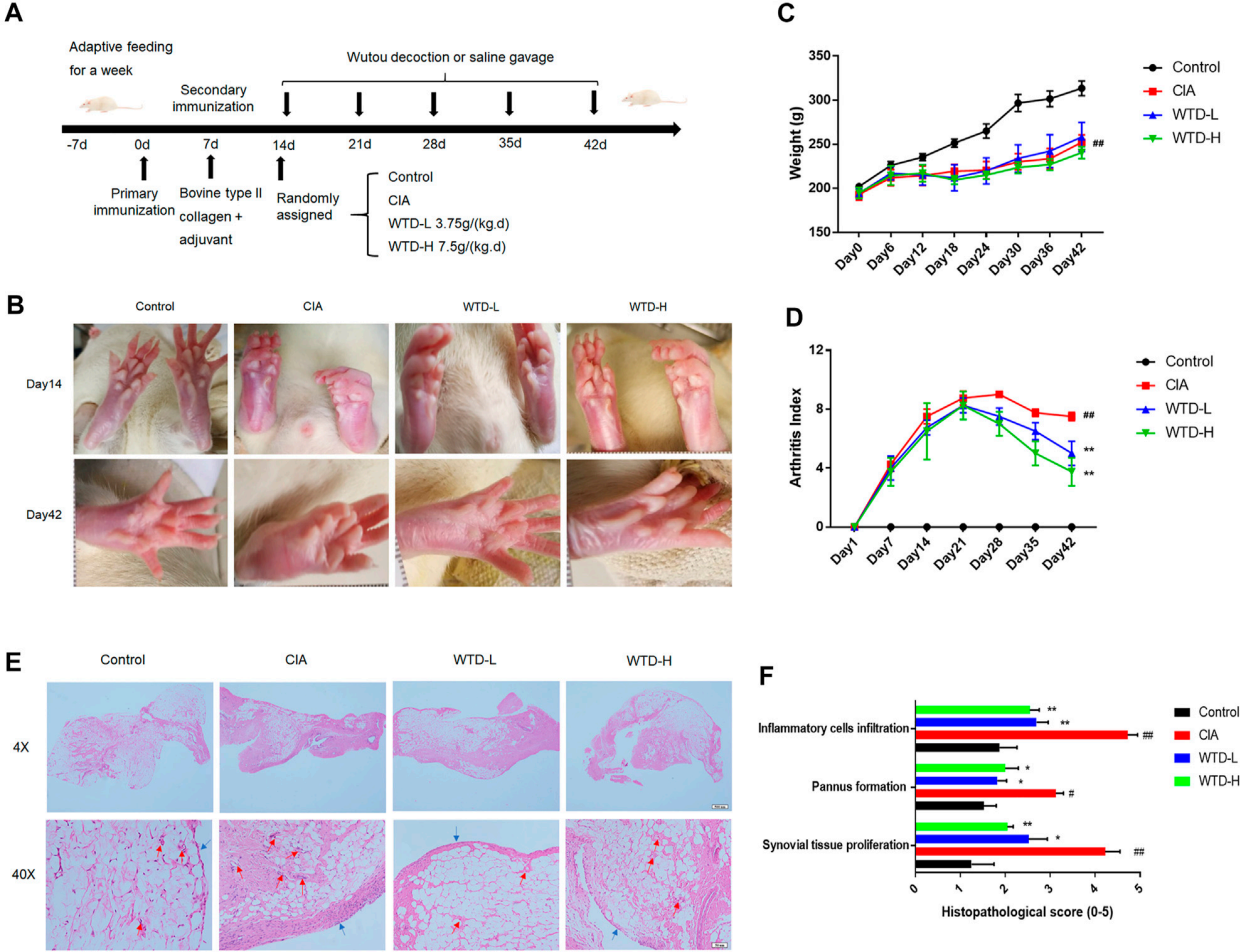
WTD treatment ameliorated CIA rats. **(A)** Schematic of CIA rat model induction and WTD intervention. **(B)** Representative images of the hind paws from the different groups. **(C)** The weights and **(D)** arthritis indexes of CIA rats treated with PBS, WTD-L (3.75 g/kg) or WTD-H (7.5 g/kg) were monitored every 6 days. **(E)** The synovium of the knee joint was sectioned for hematoxylin-eosin staining. Representative joint tissue sections are shown (original magnification, ×40 and ×200). **(F)** The histology scores of synovial tissue proliferation, pannus formation, and inflammatory cell infiltration. The results were expressed as mean ± SE. *N* = 6. #*p* < 0.05, ##*p* < 0.01 versus the control group; *p < 0.05, **p < 0.01 versus the CIA group. CIA, The Collagen-induced arthritis group; WTD-L, the low dose Wutou decoction group (3.75 g/kg/day); WTD-H, the high dose Wutou decoction group (7.5 g/kg/day).

To verify the efficacy of WTD against RA, a CIA rat model, which is widely used to clarify the mechanisms of RA and to explore potential therapeutic targets, was established and the rats were treated with two dosing regimens of WTD (Figure 2A). The body weights, arthritis scores, and synovial H&E analysis results were used to evaluate the treatment effects of WTD in CIA rats. Compared with the control group, the limbs of rats in the treated and model groups were obviously swollen on day 14 (Figures 2B,C). After 28 days of WTD treatment, the joint swelling in the treatment group was significantly improved (Figure 2B), and the arthritis score decreased (Figure 2C). The body weights of the rats decreased and could not be changed by WTD treatment (Figure 2D). From the results of H&E staining, the model group lining layer slide was significantly thickened (shown as blue arrowheads), and the number of small blood vessels (shown as red arrowheads) increased compared to the control group. Synovial hyperplasia and angiogenesis in the WTD treatment group were significantly improved.

### Wutou decoction Inhibited Angiogenesis in the Synovial Tissue of Collagen-Induced Arthritis Rats

**FIGURE 3:**
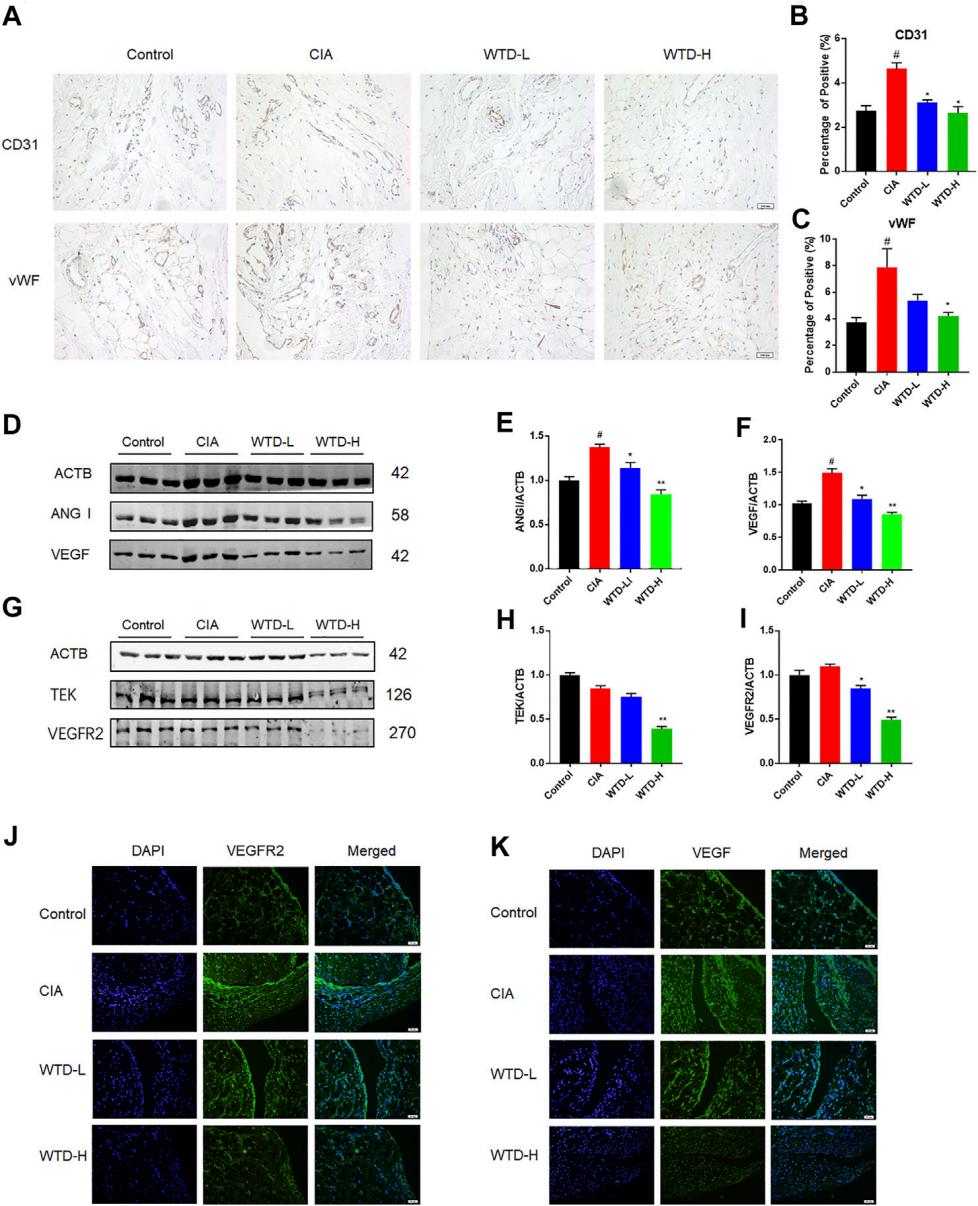
WTD inhibited angiogenesis in the synovial tissues of CIA rats. **(A)** Representative immunohistochemical staining for CD31 and vWF in synovial sections. Scale bar: 100 μm. **(B–C)** Quantitative analysis of immunohistochemical staining for CD31 and vWF in synovial tissue. **(D)** Representative western blots showing VEGF and ANGI after WTD treatment. **(E–F)** Quantitative analysis of VEGF and ANGI. **(G)** Representative western blots showing VEGFR2 and TEK after WTD treatment. **(H–M)** Quantitative analysis of the VEGFR2 and TEK western blots after WTD treatment. **(N–O)** Representative immunofluorescence staining of VEGF and VEGFR2 in synovial sections. Scale bar: 50 μm. The results were expressed as mean ± SE. *N* = 6. #*p* < 0.05, ##*p* < 0.01 versus the control group; *p < 0.05, **p < 0.01 versus the CIA group. CIA, The Collagen-induced arthritis group; WTD-L, the low dose Wutou decoction group (3.75 g/kg/day); WTD-H, the high dose Wutou decoction group (7.5 g/kg/day).

Angiogenesis is a very important in the pathogenesis of RA. To study angiogenesis in CIA rats, immunohistochemical staining of synovial tissue was performed after 28 days of WTD treatment. Compared with the control group, the expression levels of CD31 and vWF were upregulated in the model group. However, WTD treatment at either the low or high dose significantly reduced the expression of both CD31 and vWF (Figures 3A–C), which have been identified as vascular markers (Latacz et al., 2020). In addition to detecting vascular hyperplasia, we also determined the expression of vascular generation factors and their corresponding receptors in the synovial membrane by immunofluorescence and western blot analyses. Compared to the model group, the expression of VEGF and ANG1 (Figures 3F–H), as well as their related receptors (VEGFR2 and TEK) (Figure 3M–O), was downregulated in the treatment group by western blot. The effects of WTD high-dose treatment on the inhibition of angiogenesis was better than that of the low-dose group. The immunofluorescence results further verified the above conclusions. These results suggested that WTD might ameliorate angiogenesis in CIA rats by reducing vascular generation factors in synovial tissue.

### The Effects of Wutou decoction on the Proliferation, Apoptosis, Migration, and Invasion of MH7A Cells

**FIGURE 4:**
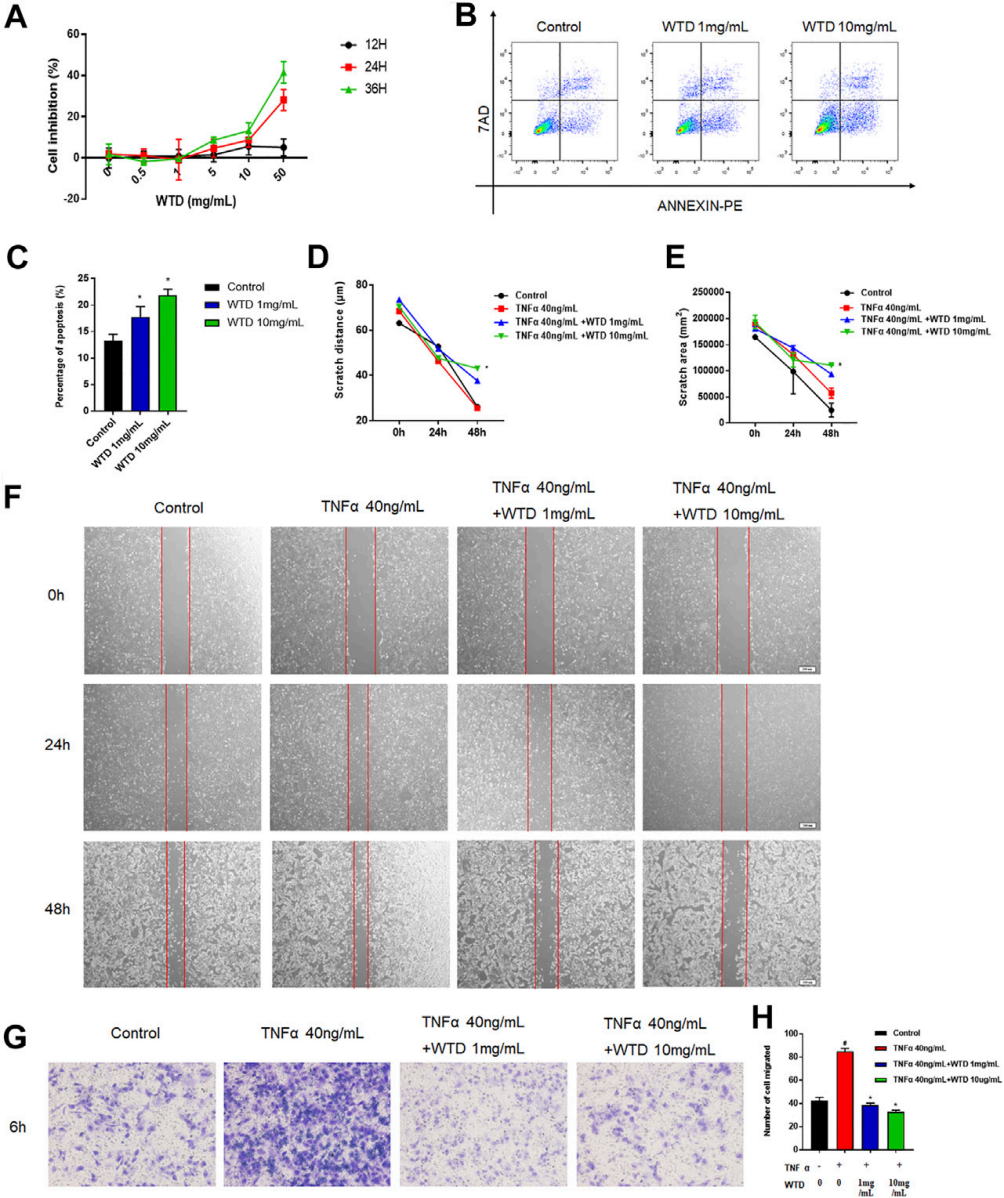
The effects of WTD on the proliferation, apoptosis, migration and invasion of MH7A cells. **(A)** Effects of WTD intervention on MH7A cell proliferation at 12, 24, and 36 h **(B–C)** Effects of MH7A cell apoptosis after WTD intervention. **(D)** Effects of MH7A cell migration at 0, 24, and 48 h after WTD intervention. Statistical diagram of € scratch distance and **(F)** scratch area. **(G)** Effects of MH7A cell invasion after WTD intervention. **(H)** Statistical results of the number of migrated cells. The results were expressed as mean ± SE. All experimental results were repeated three times. #*p* < 0.05, ##*p* < 0.01 versus the control group; *p < 0.05, **p < 0.01 versus the TNFα 40 ng/ml group.

To explore the mechanism of WTD against RA, a series of experiments were conducted in MH7A cells that interfered with WTD treatment. Abnormal hyperplasia and apoptosis resistance in fibroblasts are the main causes of synovial hyperplasia. In this study, we explored the role of WTD in MH7A cell proliferation and apoptosis. The cell proliferation experiment found that when the concentration of WTD was greater than 1 mg/ml, inhibition of MH7A cells began to be seen, exhibiting dose- and time-dependence (Figure 4A). According to the cell proliferation results, 1 mg/ml and 10 mg/ml WTD were selected for subsequent experiments. Flow cytometry detection found that WTD could promote apoptosis in MH7A cells (Figures 4B,C). Due to their migratory and invasive characteristics, FLSs can migrate to the sublining layer of the synovial membrane, where they promote angiogenesis. TNFα promoted MH7A cell migration, but WTD intervention inhibited MH7A cell migration, as shown by the cell scratch experiments (Figures 4D–F). Furthermore, WTD intervention inhibited MH7A cell invasion induced by TNFα (Figures 4G,H).

### The Effects of Wutou decoction on the Ability of MH7A Cells to Induce Human Umbilical Vein Endothelial Cell Angiogenesis

**FIGURE 5:**
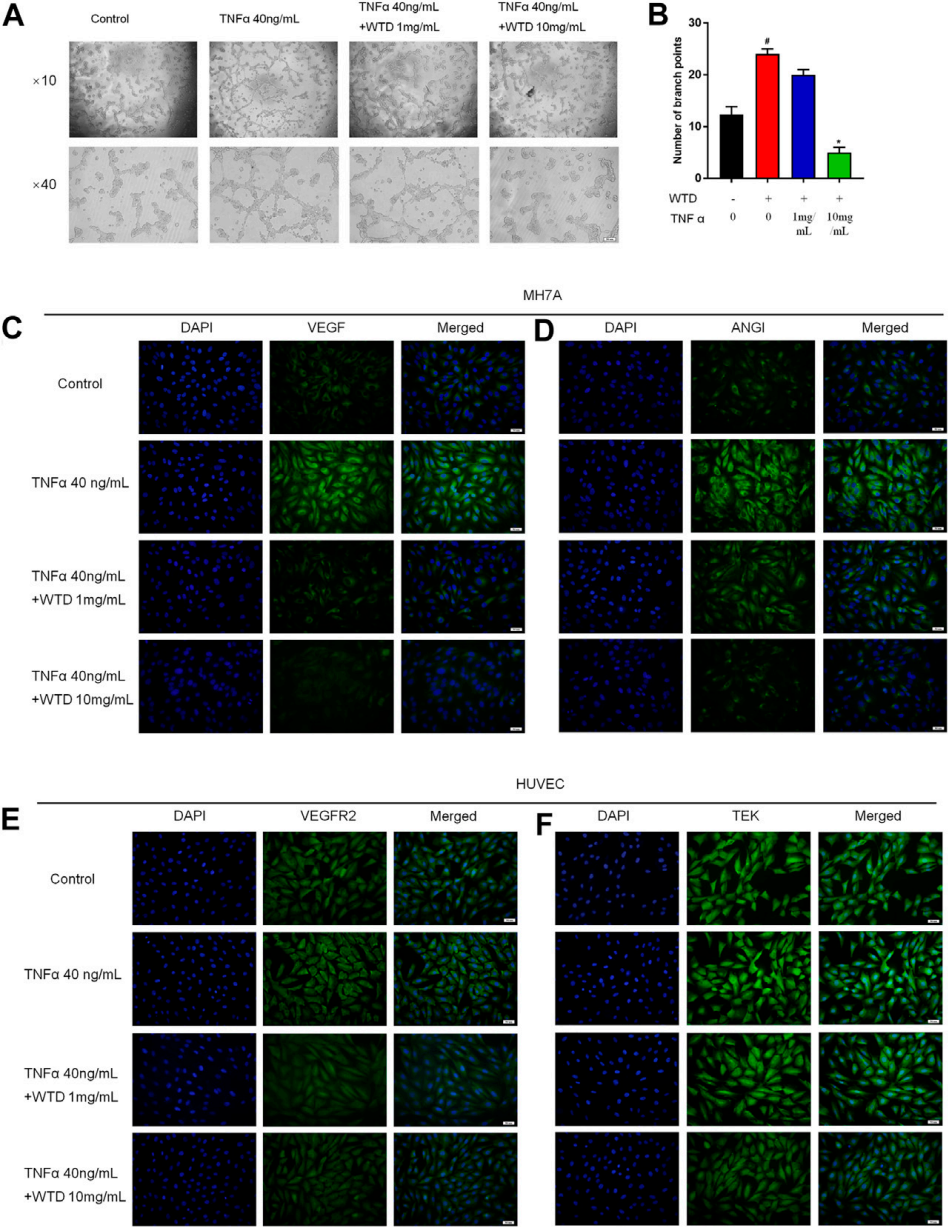
The effects of WTD on the ability of MH7A cells to induce HUVEC angiogenesis. **(A)** Representative HUVEC tube formation sections after 6 h of cocultivation with MH7A cells (original magnification,×100 and ×400). **(B)** Quantification of the number of branch points for **(A)**. **(C–D)** Representative immunofluorescence staining for VEGF and ANGI in MH7A cell sections. Scale bar: 50 μm. **(E–F)** Representative immunofluorescence staining for VEGFR2 and TEK in HUVEC sections. Scale bar: 50 μm. The results are expressed as mean ± SE. All experimental results were repeated three times. #*p* < 0.05, ##*p* < 0.01versus the Control group; *p < 0.05, **p < 0.01 versus the TNFα 40 ng/ml group.

Blood vessels are mainly composed of vascular endothelial cells and pericyte cells. However, vascular hyperplasia is affected by inflammatory factors and proangiogenic factors, which are secreted by the surrounding cells. To further confirm the mechanism by which WTD affects synovial angiogenesis, MH7A cells and HUVECs were cocultured in Transwell chambers, and HUVEC tube formation was detected after WTD was used to treat MH7A cells. The tube formation experiment showed that the ability of MH7A cells to induce HUVEC tube formation significantly increased after TNFα stimulation, and WTD reduced the ability of MH7A cells to induce HUVEC tube formation (Figures 5A,B). Furthermore, WTD inhibited the expression of the proangiogenic factors VEGF and Ang1 (Figures 5C,D) produced by MH7A cells. The expression of the corresponding homologous receptors VEGFR2 and TEK in HUVECs also decreased (Figures 5E,F). From these results, we concluded that WTD could suppress the expression of proangiogenic factors in FLSs and inhibit angiogenesis in synovial tissue.

### Network Pharmacology Analysis Shows That Wutou decoction Exerts Its Antiangiogenic Effects Through the PI3K-AKT-mTOR Pathway

**FIGURE 6:**
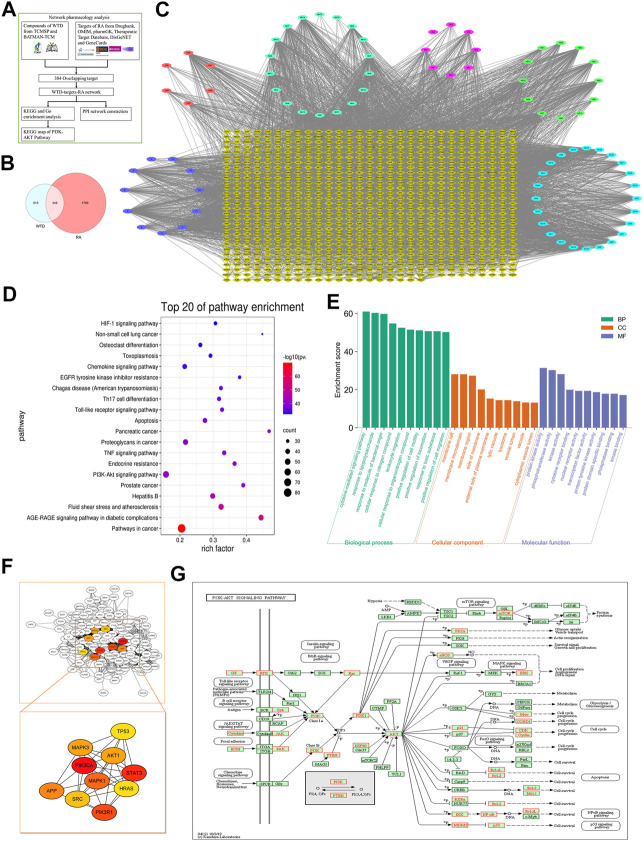
Network pharmacology analysis indicated that WTD could exert antiangiogenic effects through the PI3K-AKT-mTOR pathway. **(A)** Network Pharmacology analysis flowchart of WTD-regulated RA. **(B)** Venn diagram showing that 348 targets overlapped between WTD and RA, which were selected as the WTD-regulated RA targets. **(C)** The compound-target network was established by Cytoscape 3.8 software; the blue ellipse represents *Glycyrrhiza Radix Preparata,* the light *blue* ellipse represents *Ephedrae Herba,* the pink ellipse represents *Paeoniae Radix Alba,* the red ellipse represents *Aconiti Radix Cocta*, the green ellipse represents *Astragali Radix*, the purple ellipse represents the ingredients shared with two or more herbs, and the yellow diamonds represent WTD-regulated RA targets. **(D)** KEGG enrichment analysis and **(E)** GO enrichment analysis for the 348 WTD-regulated RA targets performed by the Metascape database. **(F)** Hub targets were identified from the PPI network using the Cytoscape plug-in MCODE. **(G)** The KEGG map of WTD-regulated targets in the PI3K-AKT-mTOR pathway. RA, Rheumatoid Arthritis; WTD, Wutou decoction; CW, Chuanwu; MH, Mahuang; BS, Baishao; HQ, Huangqi; GC, Gancao; BP, Biological process; CC, Cellular component; MF, Molecular function.

To clarify the molecular mechanism underlying the effects of WTD against RA, we established a WTD-target-RA network. A total of 863 potential targets of WTD were discovered, and 2,147 genes related to RA were obtained after deleting redundant data (Figure 6A and Supplementary Table S1). A total of 863 potential targets of WTD were compared to 2147 RA-related candidate targets, and 348 overlapping targets were found (Figures 6B,C), such as PI3K, AKT, mTOR, VEGF, and HIF-1α (Supplementary Table S2). These targets show the possible mechanism of WTD against RA angiogenesis. To identify relevant pathways and functions, KEGG pathway enrichment and GO functional analyses were performed for these 348 targets using Metascape (Figures 6D,E). The enriched target proteins are mainly related to tumors, metabolic disease, immunomodulation, and angiogenesis. Moreover, the PI3K-AKT and HIF-1α signaling pathways associated with angiogenesis were enriched. These 384 targets were ranked by their degree value, and the top 10 targets, including PIK3CA and AKT1, are shown in Figure 6F. The PI3K-AKT pathway plays an important role in the regulation of cellular functions, including cell growth, proliferation, differentiation, apoptosis, and angiogenesis. This result is consistent with the PI3K-AKT pathway target map of WTD (Figure 6G) and was verified by experiments *in vitro in vivo*. Therefore, these data indicate that the therapeutic mechanism of WTD on RA may be through these signaling pathways.

### The Effects of Wutou decoction on PI3K-AKT-mTOR-HIF1α *in vivo*

**FIGURE 7:**
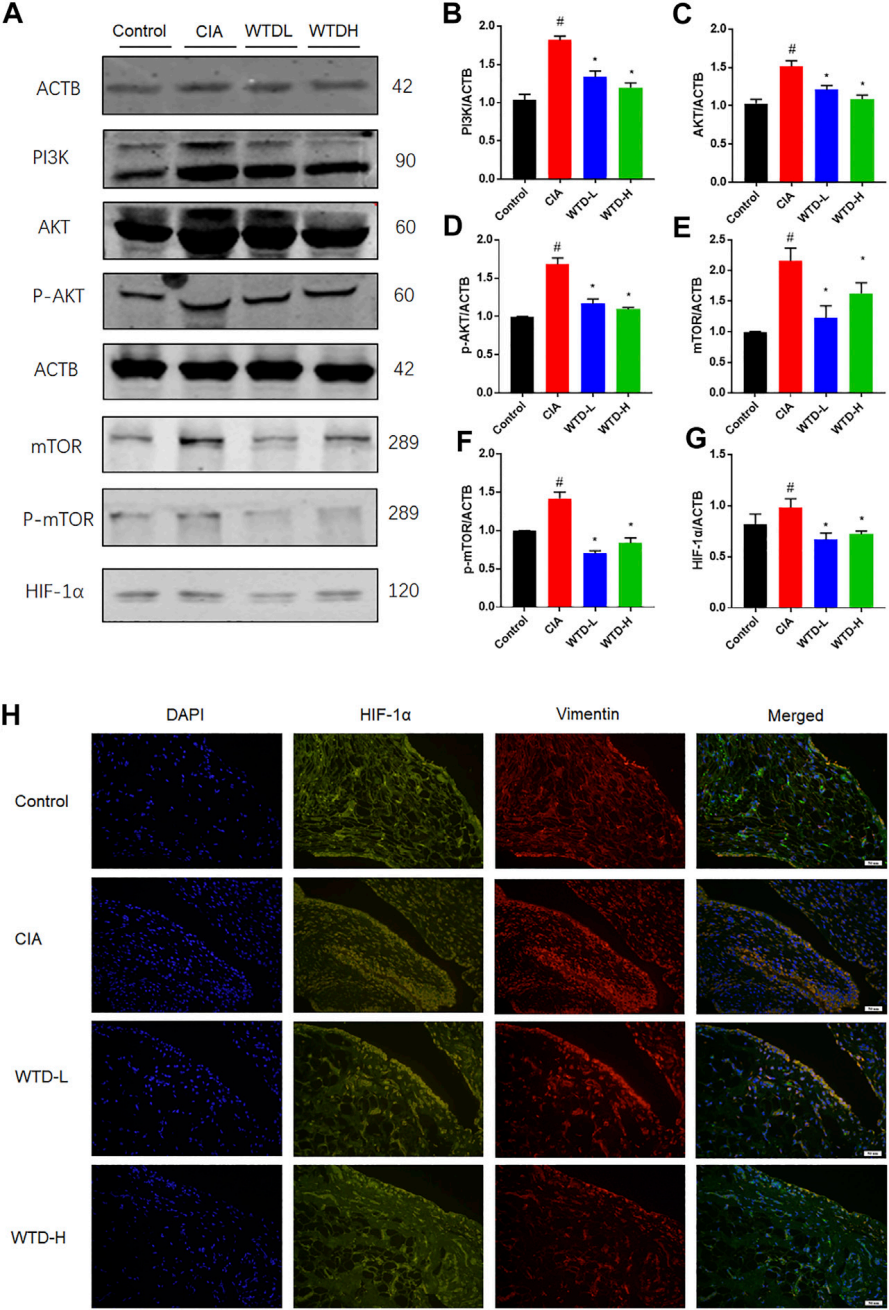
The effects of WTD on the PI3K-AKT-mTOR-HIF-1α pathway *in vivo*. **(A)** Representative western blots showing the components of the PI3K-AKT-mTOR-HIF-1α pathway after WTD treatment in CIA rats. **(B–G)** Quantitative analysis of the western blots after WTD treatment in CIA rats. **(H)** Representative immunofluorescence staining for HIF1α in the synovium of CIA rats. The results are expressed as mean ± SE. *N* = 6. #*p* < 0.05, ##*p* < 0.01 versus the control group; *p < 0.05, **p < 0.01 versus the CIA group. CIA, The Collagen-induced arthritis group; WTD-L, the low dose Wutou decoction group (3.75 g/kg/day); WTD-H, the high dose Wutou decoction group (7.5 g/kg/day). ACTB, β-actin; PI3K, Phosphatidylinositide 3-kinases; AKT, protein kinase B; p-AKT, Phosphorylated protein kinase B; mTOR, Mammalian target of rapamycin; p-mTOR, Phosphorylated Mammalian target of rapamycin; HIF-1α, Hypoxia-induced factor 1α.

After combining the of GO and KEGG analysis results, WTD was found to probably ameliorate RA through the PI3K-AKT and HIF-1α signaling pathways. Western blot analysis was used to measure the protein expression of the PI3K-AKT and HIF-1α pathways, including PI3K, AKT, p-AKT, mTOR, p-mTOR, and HIF-1α. PI3K-AKT-mTOR signaling in CIA rats was strengthened compared with the control group, and the WTD group showed inhibited PI3K-AKT-mTOR signaling compared with the CIA group. The downregulation of PI3K-AKT-mTOR signaling further led to a reduction in the HIF-1α protein expression (Figures 7A–G). Immunofluorescence verified that WTD could downregulate the expression of HIF-1α in the synovium of CIA rats (Figure 7H). Moreover, HIF-1α is an important transcription factor for the expression of pro-angiogenesis genes. Therefore, PI3K-AKT-mTOR-HIF1α could be a possible underlying mechanism by which WTD inhibits angiogenesis in the synovium of CIA rats.

### The Effects of Wutou decoction on PI3K-AKT-mTOR-HIF1α *in vitro*

**FIGURE 8:**
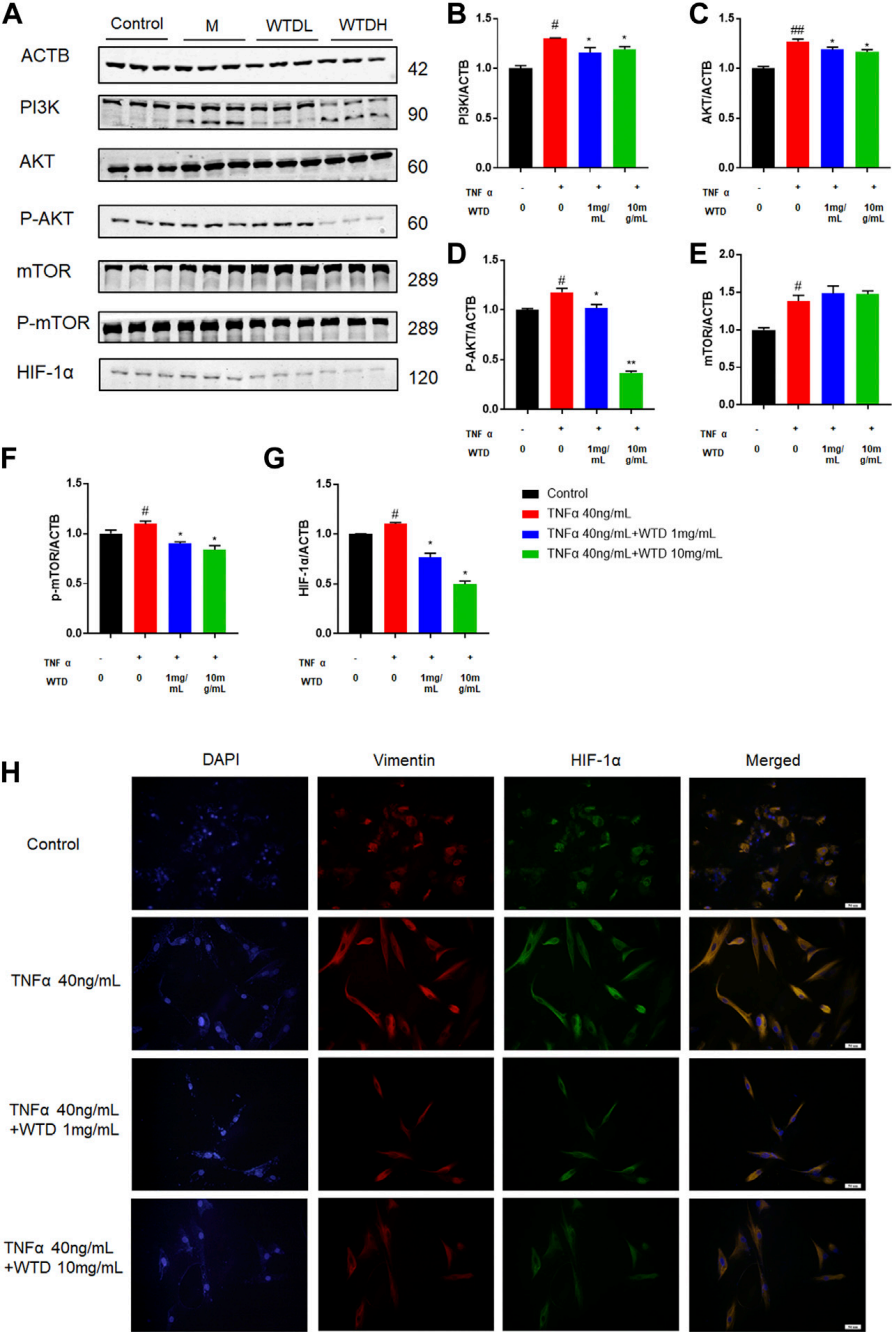
The effects of WTD on the PI3K-AKT-mTOR-HIF-1α pathway *in vitro*. **(A)** Representative western blots showing the components of the PI3K-AKT-mTOR-HIF1α pathway after WTD treatment in MH7A cells. M: TNFα 40 ng/ml; WTD-L: TNFα 40 ng/ml + WTD 1 mg/ml; WTD-H: TNFα 40 ng/ml + WTD 1 mg/ml **(B–G)** Quantitative analysis of the western blots after WTD treatment in MH7A cells. **(H)** Representative immunofluorescence staining for HIF1α in MH7A cell sections. The results are expressed as mean ± SE. #*p* < 0.05, ##*p* < 0.01versus the Control group; *p < 0.05, **p < 0.01 versus the TNFα 40 ng/ml group. ACTB, β-actin; PI3K, Phosphatidylinositide 3-kinases; AKT, protein kinase B; p-AKT, Phosphorylated protein kinase B; mTOR, Mammalian target of rapamycin; p-mTOR, Phosphorylated Mammalian target of rapamycin; HIF-1α, Hypoxia-induced factor 1α.

To further confirm the involvement of WTD in the PI3K-AKT-mTOR-HIF1α pathway, we investigated the protein expression of the abovementioned molecules in TNFα-induced MH7A cells. Notably, WTD effectively inhibited the upregulation of phosphorylated Akt and mTOR induced by TNFα *in vitro* (Figures 8A–G). Moreover, the expression of HIF-1α was inhibited in MH7A cells treated with WTD (Figure 8H). These data indicated that WTD suppressed TNFα-induced angiogenesis by downregulating PI3K-AKT-mTOR-HIF1α signaling in MH7A cells.

## Discussion

RA is a chronic systematic disease in which angiogenesis can promote inflammatory cell infiltration into the joints, leading to synovial inflammation, hyperplasia, and progressive bone destruction (Mcinnes and Schett, 2011). Currently, the main treatment for RA is nonsteroidal anti-inflammatory drugs, DMARDs and glucocorticoids (Smolen and Aletaha, 2015; Chatzidionysiou et al., 2017; Palmowski et al., 2017). However, from a long-term perspective, only DMARDs, including conventional synthetic DMARDs, biological DMARDs, and targeted synthetic DMARDs, are considered to be able to target synovial inflammation and mitigate the progression of joint damage (Buttgereit et al., 2015). In recent years, there has been great progress in the development of new antirheumatic drugs for rheumatoid arthritis, such as TNFα inhibitors, IL-6 receptor antagonists, and inhibitors targeting the JAK pathway. These new drugs have considerably delayed RA progression and improved the quality of life of RA patients (Ramiro et al., 2017). However, conventional synthetic DMARDs have liver and kidney toxicity, and biological DMARDs and targeted synthetic DMARDs have more adverse events, including the occurrence of serious infections and cancer (Ranganath et al., 2015; Silva-Fernandez et al., 2016; Pawar et al., 2019; Van Mulligen et al., 2019).

As a classic prescription, WTD has been generally used to treat Bi Zheng for two thousand years in China, and its clinical efficacy has been confirmed (Qi et al., 2014). Modern pharmacological studies have shown that WTD has anti-inflammatory effects, regulates immunity, and relieves pain. Additionally, WTD has been reported to have higher safety and no side effects compared with DMARDs, including liver and kidney toxicity, infection, and tumors. Furthermore, a variety of pharmacological studies have confirmed that ingredients in WTD were effective in the treatment of RA (Zhang et al., 2015; Guo et al., 2017). Benzoylacetone, extracted from *Aconiti Radix Cocta*, can reduce the expression of TNFα, IL-1β, and NF-κB-p65 in activated macrophages to inhibit NF-κB signaling pathways (Gai et al., 2020). Glycyrrhizin was extracted from *Glycyrrhiza Radix Preparata*, which has been proven to regulate high mobility group 1 protein, Beclin-1, and nuclear factor—inflammatory improvement factor 2, thus improving articular inflammation (Shafik et al., 2019). Glycyrrhizic acid is also the main bioactive component of *Glycyrrhiza Radix Preparata*, which has been reported to inhibit articular inflammation (Qu et al., 2019). Total glucosides of peony and total glycosides extracted from the dry roots of *Paeoniae Radix Alba* are widely used to treat RA due to their anti-inflammatory, immunomodulatory and analgesic effects (Xu et al., 2018; Zhai et al., 2018; Zhang and Wei, 2020). In addition, paeoniflorin and Talatizinine, the main ingredients of *Paeoniae Radix Alba*, could be combined with PPAR-γ, C-JUN, MMP13, and TGF-β1, which are candidate targets of the mechanism of WTD against RA (Mao et al., 2020). Collectively, these studies show that WTD or its ingredients have obvious anti-inflammatory, immunomodulatory, and analgesic effects, which may be an important in the mechanism of RA treatment by WTD. However, little is known about the role of WTD in the regulation of synovial angiogenesis.

Angiogenesis is crucial for the pathogenesis of arthritis in RA. The angiogenesis process includes endothelial cell migration, sprouting, and tube formation, followed by the wrapping of pericyte cells around the tube to promote blood vessel stability. In this process, various cytokines participate, such as proangiogenic factors, proinflammatory cytokines, matrix metalloproteases, and adhesion molecules (Elshabrawy et al., 2015). The process of angiogenesis begins with growth factors, such as VEGF, and its combination with its homologous receptor VEGFR2 on endothelial cells to stimulate the proliferation, migration, and sprouting of endothelial cells. Then, the blood vessels are stabilized by the factors generated, such as ANG1, and pericyte cells are incorporated into the newly formed substrate film to promote the blood flow process (Ferrara, 2004; Heinolainen et al., 2017). Single-cell spatial transcription group sequencing has indicated that FLSs are distributed with a spatial gradient distribution within the blood vessels in the synovial membrane (Wei et al., 2020). A variety of cellular activation processes, including cell migration and invasion, are involved in tube formation (Zhu et al., 2020). RA-FLSs exhibit aggressive features, including excessive proliferation, apoptotic resistance, and high invasion, and have long been considered to be an attractive treatment target. Activated RA-FLSs can not only migrate and invade adjacent unaffected joints, causing degradation of the articles, but also migrate to the lower layer of the synovial lining to promote angiogenesis and exacerbate synovial hyperplasia and bone destruction (Ma et al., 2019; Zhang et al., 2019). Our study shows that 10 mg/ml WTD can inhibit RA-FLS proliferation, migration, and invasion and promote apoptosis. Moreover, WTD improved CIA rat joint synovial hyperplasia and relieved joint swelling. This is consistent with the results of a previous study (He et al., 2018). Additionally, we focused on the role of WTD in the regulation of synovial angiogenesis. MH7A cells were cocultured with HUVECs, and MH7A cells secreted the hypoxia-induced factor HIF-1α after TNFα stimulation, which generated VEGF and Ang1. Additionally, the expression levels of the corresponding homologous receptors VEGFR2 and TEK in HUVECs were upregulated. FLSs can facilitate synovial angiogenesis through VEGF-VEGFR2 and then further promote blood vessel stability by Ang1-TEK. Tube formation assays in HUVECs further proved that WTD can inhibit MH7A cell-induced angiogenesis. In addition, *in vivo* experiments confirmed that FLS-induced synovial vascular hyperplasia can be blocked by WTD, but the specific mechanism is not clear. Determination of the potential mechanism of Chinese herbal medicines can not only discover the potential goals of disease treatment but also clarify the effective ingredients in the Chinese herbal medicines to provide a scientific basis for their clinical use.

With the development of bioinformatics, modern network pharmacology is considered to be a very important tool to explore the complex mechanism of TCM compounds (Ru et al., 2014). This study identified the main components of WTD by querying the traditional Chinese medicine database TCM-SP and collecting the main targets. Combined with modern pharmacological study of RA disease treatment targets and the targets of WTD, the main KEGG pathways of WTD in the treatment of RA include the AGE-RAGE signaling pathway, PI3K-AKT signaling pathway, TNF signaling pathway, Toll-like receptor signaling pathway, and HIF-1α signaling pathway.

**FIGURE 9:**
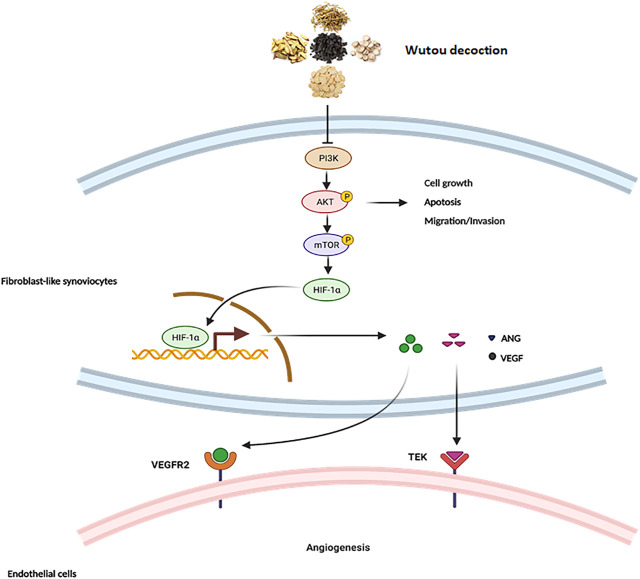
The mechanism of WTD against angiogenesis in RA. Overview of the underlying mechanisms of WTD treatment by the PI3K/AKT/mTOR signaling pathway. Activation of PI3K by receptor tyrosine kinases promotes cell growth, proliferation, migration and invasion as well as angiogenesis. PI3K signaling can activate and phosphorylate AKT, and p-AKT can inhibit the expression of apoptosis-related genes to allow the cells to survive while promoting the expression of genes related to cell proliferation, migration, and invasion. More importantly, p-AKT can activate mTOR to upregulate the expression of HIF1α. HIF-1α is stabilized under hypoxic conditions and quickly translocated to the nucleus, where it facilitates the expression of the associated genes VEGF and ANGI). However, VEGF and ANGI can bind corresponding homologous receptors coupled to endothelial cells to promote synovial angiogenesis. PI3K, Phosphatidylinositide 3-kinases; AKT, protein kinase B; mTOR, Mammalian target of rapamycin; HIF-1α, Hypoxia-induced factor 1α; VEGF, Vascular endothelial growth factor; VEGFR2, Vascular endothelial growth factor receptor 2; ANG1, Angiopoietin 1; TEK, Angiopoietin-1 receptor.

The PI3K-AKT pathway plays important roles in cell proliferation, apoptosis, and differentiation (Karar and Maity, 2011). In this study, we found that WTD can inhibit MH7A cell proliferation and promote MH7A cell apoptosis. WTD can also inhibit the invasion and migration of activated MH7A cells induced by TNFα. However, further experiments are needed to verify whether WTD affects the PI3K-AKT pathway to inhibit MH7A cell proliferation and reduce apoptosis resistance. Additionally, we focused on the role of WTD in angiogenesis in the synovial tissues during RA. Many inhibitors targeting the PI3K-AKT pathway have shown the effects of downregulating the expression of VEGF and suppressing angiogenesis. The PI3K-AKT pathway plays an important role in regulating angiogenesis in RA. The connection between the PI3K-AKT pathway and angiogenesis has been confirmed in many studies. Ginsenoside RG1 facilitates cerebrovascular generation and increases ischemic stroke by increasing the expression of VEGF through the PI3K-AKT-mTOR signaling pathway (Chen et al., 2019). Shikonin (SKN), the main chemical component separated from zicao (*Lithospermum erythrorhizon Sie*b), has an antiangiogenic effect by impeding the PI3K-AKT signaling pathways in RA. Artesunate can suppress inflammation and prevent cartilage and bone destruction in collagen-induced arthritis models in rats (Ma et al., 2019). Many ingredients in WTD have also been confirmed to have pharmacological effects within the PI3K-AKT pathway (Liu et al., 2020). Studies have shown that glycyrrhizenic acid is produced by adjusting autophagy and inhibiting the PI3K/AKT/mTOR pathway after induction by inflammatory factors in ALI, which can provide new therapies for Ali GA (Qu et al., 2019). Studies have shown that astragalus IV (AS-IV) treatment promotes cell proliferation and tube formation and induces activation of the PTEN/PI3K/AKT signaling pathway (Cheng et al., 2019). Paeoniflorin can inhibit mesangial cell proliferation and the inflammatory response through the PI3K/Akt/GSK-3β pathway. Inflammation or hypoxic stimulation results in the expression and stabilization of HIF-1α, increasing vascular endothelial growth factor expression (VEGF) (Park et al., 2007). Is there a similar mechanism by which WTD ameliorates angiogenesis in RA? The PI3K/AKT pathway can increase VEGF secretion through both hypoxia-induced factor 1 (HIF-1)-dependent and HIF-1-independent mechanisms (Karar and Maity, 2011; Li et al., 2018; Chen et al., 2019; Zhu et al., 2020). In this study, WTD interrupted the PI3K-AKT pathway and suppressed HIF-1α expression. Therefore, the PI3K-AKT-mTOR-HIF-1α pathway could be the underlying mechanism by which WTD ameliorates angiogenesis in RA (Figure 9).

There are some limitations in of this study. The concentration of the WTD extract used in this experiment was 1–10 mg/ml, which seems to have no pharmacological significance. However, the compound ingredients of traditional Chinese medicine are complicated. This is a total concentration of a mixed drug not a concentration of a chemical component. According to the quantification of fingerprint, the content of the main components of WTD we calculate (supplement 4) is pharmacological significance.

## Conclusion

In conclusion, we demonstrated that WTD could treat RA by inhibiting MH7A cell proliferation, migration, invasion, and promotion of angiogenesis. The underlying mechanism by which WTD ameliorated angiogenesis in RA was associated with the inhibition of the PI3K-AKT-mTOR-HIF1α signaling pathway. This experiment can provide a scientific explanation for the clinical applications of WTD and facilitate the discovery of natural drugs for treating RA. Furthermore, it may complement existing RA drug treatments from the viewpoint of angiogenesis rather than suppressing inflammation and regulating immunity. This experiment suggests WTD could be considered as a promising alternative candidate for RA characterized by joint swelling and synovial vascular hyperplasia.

## Data Availability

The original contributions presented in the study are included in the article/Supplementary Material, further inquiries can be directed to the corresponding author.
